# Conventional Type 1 Dendritic Cells in Intestinal Immune Homeostasis

**DOI:** 10.3389/fimmu.2022.857954

**Published:** 2022-05-27

**Authors:** Izumi Sasaki, Takashi Kato, Hiroaki Hemmi, Yuri Fukuda-Ohta, Naoko Wakaki-Nishiyama, Asumi Yamamoto, Tsuneyasu Kaisho

**Affiliations:** ^1^ Department of Immunology, Institute for Advanced Medicine, Wakayama Medical University, Wakayama, Japan; ^2^ Laboratory of Immunology, Faculty of Veterinary Medicine, Okayama University of Science, Ehime, Japan

**Keywords:** XCR1, intestine, gene targeting, T cell, dendritic cell, subset

## Abstract

Dendritic cells (DC) play critical roles in linking innate and adaptive immunity. DC are heterogenous and there are subsets with various distinct functions. One DC subset, conventional type 1 DC (cDC1), can be defined by expression of CD8α/CD103 in mice and CD141 in humans, or by expression of a chemokine receptor, XCR1, which is a conserved marker in both mice and human. cDC1 are characterized by high ability to ingest dying cells and to cross-present antigens for generating cytotoxic CD8 T cell responses. Through these activities, cDC1 play crucial roles in immune responses against infectious pathogens or tumors. Meanwhile, cDC1 involvement in homeostatic situations is not fully understood. Analyses by using mutant mice, in which cDC1 are ablated *in vivo*, revealed that cDC1 are critical for maintaining intestinal immune homeostasis. Here, we review the homeostatic roles of cDC1, focusing upon intestinal immunity.

## Introduction

The intestinal immune system responds to pathogenic organisms in a protective manner. Meanwhile, it also prevents responses against a variety of commensal resident bacteria or viruses and dietary components. Intestinal immunity should therefore be delicately tuned to maintain its homeostasis. Intestinal immune homeostasis depends on intimate interactions between innate and adaptive immune cells, epithelial cells and commensal microorganisms (1). Dysregulation of this homeostasis could lead to a variety of inflammatory bowel diseases and also to systemic inflammatory or metabolic diseases. Elucidation of the molecular and cellular mechanisms of how intestinal immune homeostasis is regulated is therefore of great importance.

The intestine contains a variety of immune cells, including B and T lymphocytes and innate immune cells such as macrophages or dendritic cells (DC). DC are professional antigen presenting cells (APC) that can produce a variety of cytokines in response to innate immune sensor signaling and that can support activation and differentiation of T cells (2). DC are heterogeneous and several subsets have subset-specific functions (3, 4). One subset of DC, conventional type 1 DC (cDC1), have recently been in the spotlight for its critical roles in anti-tumor immunity and cancer immunotherapy (5, 6). Meanwhile, it is unclear whether cDC1 play certain roles in homeostatic conditions, although retinoic acid, a metabolite of vitamin A derived from the diet, is involved in regulation of intestinal homeostasis by affecting a variety of immune cells including subsets of DC (7). cDC1 have been shown to be critically involved in maintaining intestinal immune homeostasis (8–10). Here, we review the homeostatic roles of cDC1 in intestinal immunity.

## DC Subsets and Their Ablation *In Vivo*

### DC Subsets

DC include plasmacytoid DC (pDC) and cDC. In mice, pDC can be defined by several pDC-specific markers such as BST-2 or Siglec-H and is characterized by the ability to produce large amounts of type I interferons (IFNs) following detection of viral nucleic acids through binding to TLR7 or TLR9 (11). Also in humans, pDC exist as a type I IFN-producing cell in response to TLR7/9 signaling. pDC are involved not only in antiviral immunity, but also in the pathogenesis of certain autoimmune diseases such as systemic lupus erythematosus or psoriasis (12).

In mice, cDC are a major population of DCs and can be further divided according to the expression of CD8α and CD11b. CD103 can also be used instead of CD8α. In the spleen or dermal lymph nodes, DC consist mainly of two subsets, CD103+CD11b- DC and CD103-CD11b+ DC, corresponding with cDC1 and cDC2, respectively. cDC1 were originally identified as CD8α+ DC and recognized for high crosspresenting activity, through which exogenous antigens incorporated by endocytosis or phagocytosis are presented with MHC class I to generate cytotoxic T cell responses (13). cDC1 also have notable potent ability to incorporate dying cells and produce proinflammatory cytokines. The generation of cDC1 depends on transcription factors such as IRF8 and BATF3 (14–16). cDC2 are more heterogenous than cDC1 and supports antigen presentation with MHC class II to generate various types of Th responses, including Th1, Th2 or Th17 responses, depending on situations (17–25). Involvement of transcription factors for cDC2 generation is complicated. For example, cDC2 can be divided into at least two subsets according to differential involvement of Notch2 and Klf4 in cDC2 generation (21, 23). IRF4 is highly expressed in cDC2, but is only partially involved in cDC2 generation, although it is critical for migration of cDC2 from skin or intestine to the draining lymph nodes (19, 20, 26, 27).

In humans, DC can be defined in the peripheral blood as MHC class II+ leucocytes that lack surface expression of lineage markers for T cells (CD3), B cells (CD19), and monocytes (CD14) (28). CD141 (thrombomodulin, BDCA-3) + DC correspond with cDC1, based on the crosspresenting activity and gene expression pattern (28). CD1c+ DC are similar to cDC2, although their similarity is much less than that between murine cDC1 and human CD141+ DC (28). In addition to these DCs, both humans and mice have monocyte-derived inflammatory DC (28).

### *In Vivo* Ablation of cDC1

Specific ablation of subsets of DC has provided useful information on their *in vivo* roles (29). For cDC1, specific ablation was observed in mutant mice lacking a transcription factor, BATF3, also known as Jun dimerization protein p21SNFT, which is expressed abundantly in cDC, including cDC2, albeit not as highly as in cDC1 (16). Mutant mice have been widely used as cDC1-ablated mice and studies on BATF3-deficient mice clarified that cDC1 are critically involved in protective immunity against viral or bacterial infection and tumors (16, 30–35). Meanwhile, it should be noted that pathogen infection or IL-12 administration can restore cDC1 in BATF3-deficient mice (32).

cDC1 ablation can also be achieved also by genetically manipulated mice, in which cDC1-specific genes were targeted. A gene for a chemokine receptor, XCR1, is a representative cDC1-specific gene (36, 37). XCR1-DTR Venus mice were generated by knocking the gene for a fusion protein consisting of human diphtheria toxin receptor (DTR) and a fluorescence protein, Venus, into the *Xcr1* locus (**Table 1**) (38). XCR1+DC can be ablated transiently in XCR1-DTR Venus mice, through which a variety of immune responses have been analyzed (33, 38, 48–57). Mutant mice lacking XCR1+ DC constitutively were also generated by crossing mutant mice carrying a gene encoding cre recombinase in the *Xcr1* locus and R26:lacZbpA^flox^-DTA mice (8, 58). Murphy et al. also generated knock-in mice carrying the cre recombinase gene with a gene for a fluorescence protein, mCherry in the *Xcr1* locus (40). In the mice, XCR1+DC can be monitored by mCherry expression. Malissen et al. also generated another mutant mouse, in which genes encoding cre recombinase and monomeric teal fluorescent protein 1 (mTFP1) were knocked into the *Xcr1* locus (41). In the mice, XCR1+DC can be monitored by mTFP1 expression and endogenous *Xcr1* expression is retained due to the effect of an internal ribosomal entry site. Furthermore, mutant mice carrying the *DTR* or cre recombinase gene casettes in the locus of other cDC1-specific genes such as *Clec9A* or *a530099/19rik (Gpr141b/Karma)* were also generated (39, 44–47).

**Table 1:** Knock-in mice for analyzing XCR1+ DC.

Targeted gene locus	Knocked-in gene	Purpose	Ref
*Xcr1*	DTR Venus	Inducible ablation	(38)
		Marking	
	cre	Gene deletion	(8, 39)
	cre, mCherry	Gene deletion	(40)
		Marking	
	cre, mTFP1	Gene deletion	(41)
		Marking	
	Venus	Marking	(38)
	GFP	Marking	(42)
	KikGR	Marking	(43)
		(Photoconvertible)	
*Clec9a*	DTR	Inducible ablation	(44)*
	Cre	Gene deletion	(45)
	GFP	Marking	(46)
*Karma*	DTR, tdTomato**	Inducible ablation	(47)
		Marking	
	Cre	Gene deletion	(39)

*This mutant was generated by transfection with a recombineered bacterial artificial chromosome (BAC) clone carrying DTR in the Clec9a locus.

**tdTomato represents the fluorescent tandem dimer Tomato.

Other reporter mice for monitoring XCR1+ DC are also shown (**Table 1**). Fluorescence proteins used for monitoring include Venus, green fluorescence protein (GFP), and Kikume Green-Red (KikGR) (42, 43). KikGR is a photoconvertible fluorescence protein that can turn red from green when illuminated with a blue light and can be used for tracking cell migration (43).

These mice are useful for clarifying the *in vivo* roles and behavior of XCR1+ DC.

## Critical Roles of XCR1+ DC in the Intestine

In the intestine or mesenteric lymph nodes, CD103+CD11b+ DC exist as a major subset and intestinal CD11c+ cells consist of three subsets, including CD103+CD11b-, CD103+CD11b+, and CD103-CD11b+ cells. CD103+CD11b+ cells are closely related to cDC2 in terms of the gene expression profile and their dependency on IRF4. CD103^+^CD11b^+^ cells are involved in Th17 or regulatory T (Treg) cell differentiation (19, 20, 59, 60) and critical for antifungal immunity (61). CD103-CD11b+ cells include macrophages and may be involved in regulating inflammation by producing anti-inflammatory cytokines such as IL-10 or TGF-β (62, 63). CD103+CD11b- cells are quite similar to cDC1 in other tissues in terms of gene expression profiles and their dependence on IRF8 and BATF3. Earlier studies on the intestine have analyzed CD103+CD11c+ and CD103-CD11c+ DC (59, 60). However, it was not clear whether or how a minor population, CD103+CD11b-CD11c+ cells, i.e. cDC1, are involved in intestinal immunity.

Constitutive ablation of cDC1 with the retention of other DC subsets such as CD103^+^CD11b^+^ as well as CD103^-^CD11b^+^ cells was achieved in XCR1-DTA and DC-specific IRF8-deficient mice (8, 9, 64). In those mice, lamina propria (LP) T cells in the small intestine were decreased. Significant numbers of lymphocytes reside within the intestinal epithelium and are known as intraepithelial lymphocytes. Intraepithelial T cells include not only conventional CD4 or CD8 T cells expressing TCRαβ but also T cells expressing TCRγδ or both CD4 and CD8. A subpopulation of such intestine-specific T cells expresses a CD8 homodimer, CD8αα, instead of a CD8 heterodimer, CD8αβ, that conventional CD8 T cells have. In addition to LP T cells, both conventional and intestine-specific T cell subsets in IEL were decreased in mice with constitutive ablation of cDC1.

Importantly, T lineage cell numbers were retained in other tissues, including the thymus, spleen, and dermal and mesenteric lymph nodes, lung and large intestine (8, 9). Thus, although cDC1 were deleted in the whole body, T cell decrease was detected specifically in the small intestine. Intestinal T cells remained in XCR1-DTA mice were more prone to death than those in control mice, although their proliferative state was normal, indicating that cDC1 are not involved in proliferation, but in survival of intestinal T cells (8). Furthermore, in wildtype mice, LP T cells showed lower expression of CD62L and higher expression of CD103 than splenic or lymph node T cells. This expression pattern was less prominent in remaining T cells in XCR1-DTA than in wildtype mice (8). DC-specific IRF8-deficient mice also exhibited decreased expression not only of CD103, but also of gut homing receptors such as CCR9 or α4β7 on the intestinal T cells (9). The decreased expression of CCR9 can be ascribed to defective production of retinoic acid, which depends on aldehyde dehydrogenase activity from cDC1 (9). Concerning CD4 T cell differentiation, ablation of cDC1 led to decrease of IFN-γ and increase of IL4, IL-5, IL-17 and IL-22 expression. These results indicate skewed differentiation from Th1 towards Th2 or Th17 cells and suggest involvement of cDC1 in driving Th1 cell differentiation in the steady state. Notably, DC-specific IRF8-deficient mice also showed impaired IgG2c responses after oral administration of eggs of a gastrointestinal parasite, *Trichulis muris*, indicating that cDC1 are required for Th1 responses to the parasite (9). Thus, in the intestine, cDC1 maintain intestinal T cell homeostasis by supporting survival, expression pattern of surface molecules including gut homing receptors and Th1 differentiation of intestinal T cells.

Loss or decrease of intraepithelial T cells due to gene mutations or chemical reagents lead to severe manifestations of colitis, indicating that those T cells are involved in preventing intestinal inflammation (65–69). The immunoregulatory activity of the intraepithelial T cells depends on the production of anti-inflammatory cytokines such as IL-10. XCR1-DTA mice showed no overt signs of intestinal inflammation in the steady state. They did show exaggerated manifestations of dextran sodium sulfate (DSS)-induced colitis (8), indicating that XCR1+ DC are involved in preventing intestinal inflammations likely through the maintenance of intestinal T cell populations.

The critical roles of cDC1 in intestinal immune homeostasis were also demonstrated by transient deletion of cDC1 (10). Inducible ablation of cDC1 in Clec9A-DTR mice led to decrease of intestinal T cells and exaggeration of DSS-induced colitis. cDC1 were involved in maintaining not only IFN-γ expression from T cells, but also expression of IFN-γ inducible genes from epithelial cells. This IFN-γ-induced cascade is involved in preventing intestinal inflammation, which was verified by the findings that IFN-γ-deficient mice showed enhanced susceptibility to DSS-induced colitis and that IFN-γ induction by an immunostimulatory oligonucleotide ameliorated the severity of the colitis. This study further showed IL-12 or IL-15 from cDC1 should induce LP or intraepithelial lymphocytes to produce IFN-γ that subsequently can trigger an anti-inflammatory response in intestinal epithelial cells. At present it remains unclear how IFN-γ exerts such protective roles, although induction of an anti-inflammatory enzyme, indoleamine 2,3 dioxygenase (IDO1), which is abrogated in both cDC1-ablated and IFN-γ-deficient mice, may be involved (10).

## Molecular Mechanisms for XCR1+ DC-Dependent Intestinal Homeostasis

XCR1+ DC occupy less than 10% of intestinal DC and the other DC or macrophages fail to compensate for the functions of XCR1+ DC. This suggests that certain molecule(s) specifically expressed by XCR1+ DC play critical roles in maintaining intestinal homeostasis. XCR1 itself should be a candidate molecule. A ligand of XCR1 is XCL1 in mice and XCL1 and its close relative, XCL2, in humans. In both species, XCL1 is abundantly produced by NK cells and activated T, especially CD8 T cells. XCL1- or XCR1-deficient mice show partial defects in CD8 T cell responses during the late phase of immunization (36). XCL1 is also expressed in thymic medullary epithelial cells and critical for localization of XCR1+ DC into the thymic medulla (70). XCL1 deficiency leads to disturbance of this localization and partial defects in Treg cell generation (70). Thus, the XCR1-XCL1 axis has the potential to modulate interactions of T cells and DC. However, it was unclear, however, whether the axis regulates intestinal T cell homeostasis and how this happens.

In XCL1- and XCR1-deficient mice, intestinal, but not spleen or lymph node, T cells were significantly decreased as observed in the XCR1-DTA or DC-specific IRF8-deficient mice (8, 9, 64). The XCR1-XCL1 axis is therefore suggested to be critical for maintaining intestinal T cell populations.

It is important to consider the sources of XCL1. Upon viral infection, activated CD8 T cells are known to recruit XCR1+ DC by producing XCR1+ DC (50). In steady states, XCL1 expression level is much higher in NK cells than in CD4 or CD8 T cells in the spleen (8). However, all intestinal T cell subsets expressed higher levels of XCL1 than splenic T cells and the expression level was comparable with that of splenic NK cells. Importantly, *Xcl1* expression in LP T cells of XCR1-DTA mice was decreased to approximately 20% of that in control mice (8). XCR1+ DC in the intestine are therefore indicated to be critical for driving XCL1 expression in intestinal T cells.

T cells are stimulated by XCR1+ DC to keep the XCL1 expression level, so it is important to address whether XCR1+ DC are also stimulated by T cells. In XCR1- and XCL1-deficient mice, CD103+CD11b- DC were increased in LP, but decreased in mesenteric lymph nodes (8). In the homeostatic conditions, DC leave LP and migrates into the mesenteric lymph nodes. The phenotype of XCR1- and XCL1-deficient mice therefore suggests defects in this homeostatic migration of CD103+CD11b- DC. Consistent with this speculation, expression of CCR7, a chemokine receptor involved in migration of DC from LP to mesenteric lymph nodes (71), was decreased in XCR1-deficient CD103+CD11b- DC (8). Furthermore, increase of LP and decrease of mesenteric lymph node CD103+CD11b- DC were also observed in RAG2-deficient mice, which lack B and T lymphocytes (8), also supporting the involvement of T cells. Thus, XCR1+ DC migrate from LP to mesenteric lymph nodes by the function of the XCR1-XCL1 axis and the interaction between XCR1+ DC and T cells is bidirectional.

It is interesting why T cells are decreased in the small, but not large intestine due to loss of XCR1+ DC. Such a segment-specific T cell decrease is interesting and not surprising, because intestinal immunity is regulated in a segment-specific manner (72–74). Notably, compartmentalization and gene expression profiles of DC subsets are different among intestinal segments. Furthermore, several reports also show differential roles of DC subsets in intestinal immunity (74, 75). Further studies are necessary to clarify segment-specific roles of XCR1+ DC or other DC subsets in intestinal immunity.

## Scenario for the Interaction of XCR1+ DC and T Cells in the Intestine

**Figure 1:**
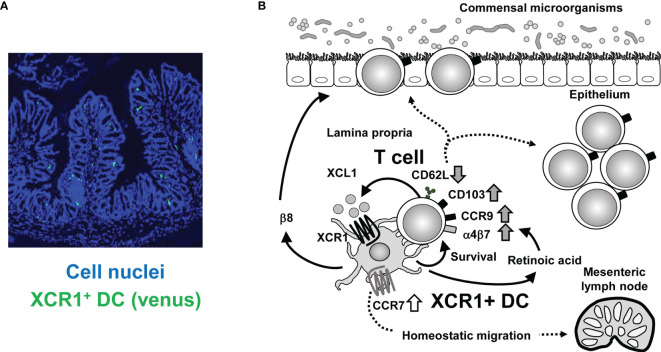
Crosstalk between XCR1+ DC and intestinal T cells. **(A)** Immunofluorescence imaging of intestinal sections from XCR1-Venus (*Xcr1^+/venus^*) mice. XCR1+ DC can be detected by Venus expression. Cell nuclei were stained with Diamidino-2-phenylindole. **(B)** Once activated, intestinal T cells produce XCL1, which attracts XCR1+ DC. XCR1+ DC support survival, upregulation of CD103, CCR9, α4β7, and XCL1 expression, downregulation of CD62L expression and generation of CD4+CD8αα+ IEL, thereby leading to maintenance of intraepithelial and LP T cell populations. T cell-derived XCL1 then keep CCR7 expression of XCR1+ DC to enable migration of XCR1+ DC from the LP to the mesenteric lymph nodes. High expression of XCR1 and β8 integrin and high activity of aldehyde dehydrogenase, which can convert retinal to retinoic acid, contribute to XCR1+DC-dependent mechanisms.

Understanding the crosstalk between XCR1+ DC and intestinal T cells may provide insight into the mechanisms that maintain intestinal homeostasis. Based on current data we propose the following model (**Figure 1**). First, intestinal T cells can be activated by any type of APC in the steady state. Once activated, T cells produce XCL1, which attracts XCR1+ DC selectively among DC subsets. Then XCR1+ DC supports T cell survival and activation, which is represented by decreased expression of CD62L and increased expression of CD103. XCR1+ DC produce retinoic acid due to their high aldehyde dehydrogenase activity, thereby increasing T cell expression of gut homing receptors such as CCR9 and α4β7 (9). XCR1+ DC also show high expression of β8 integrin, which is required for IEL generation (9). Furthermore, XCR1+ DC is involved in skewing Th cell polarization from Th2 or Th17 to Th1 cells (8, 9). High expression level of XCL1 in T cells is also driven and kept by XCR1+ DC. Meanwhile, XCR1+ DC are also stimulated by XCL1 from T cells. XCR1 signaling should lead to upregulation of CCR7 expression and promote homeostatic migration of XCR1+ DC from LP to mesenteric lymph nodes. This intimate and two-way crosstalk between XCR1+ DC and T cells through the XCR1-XCL1 axis is critical for homeostasis of intestinal immunity and prevention of aggravation of intestinal inflammation.

In germ-free mice, intraepithelial T cells are significantly decreased (76, 77). Furthermore, certain commensal bacteria, such as *Lactobacillus reuteri*, have been shown to promote generation of intraepithelial CD4+CD8αα+ T cells (78). Commensal microorganisms therefore contribute to maintenance of intestinal T cell populations and XCR1+ DC are likely involved in this step. Intriguingly, certain *Klebsiella* strains, when colonized in the intestine, induce Th1 cell differentiation in an XCR1+ DC-dependent manner (79), providing more evidence for the involvement of XCR1+ DC in intestinal T, especially Th1, cell responses.

Discussion of whether or how cDC1 are involved in Treg generation, and how this might work, is important. In BATF3-deficient, DC-specific IRF8-deficient or XCR1-DTA mice, CD4+Foxp3+ Treg cells show a normal population at the steady state in the intestine (8, 9, 80). In BATF3-deficient mice, Treg cell induction was attenuated after infection with a gastric pathobiont, *Helicobacter pylori* (81). Furthermore, in cDC-specific IRF8-deficient mice, in which *Irf8* is deleted by cre recombinase expression under the control of *Zbtb46*, a cDC-specific gene, Treg cell induction after oral administration of ovalbumin is impaired, but oral tolerance is intact (64). cDC1 are therefore dispensable for Treg cell population at the steady state in the intestine as well as for oral tolerance, although it is required for optimal Treg cell induction.

## *In Vivo* Roles of cDC1 in Various Tissues

cDC1 are present not only in the intestine but also in various peripheral tissues including skin, lung, and liver. It is interesting to clarify how cDC1 in those tissues function in homeostatic or pathogenic conditions. In the nonobese diabetic (NOD) background, autoreactive T cells were absent in islets of Langerhans and autoimmune manifestations were ameliorated in BATF3-deficient mice, indicating that cDC1 in islets are involved in the pathogenesis of autoimmune diabetes (82). In atherosclerosis, cDC1 increase in the aorta and ablation of cDC1 by deleting Flt3, a cytokine receptor, leading to increase of atherosclerotic lesion size and plaque area, concomitantly with decrease of Treg cells (83). This implies protective roles of cDC1 in the pathogenesis of atherosclerosis, although it should be kept in mind that Flt3 deletion results in decrease of cDC2 as well as cDC1. Meanwhile, in non-alcoholic steatohepatitis (NASH), DC including cDC1 increase in the liver and depletion of XCR1+ DC attenuated liver pathology, indicating that XCR1+ DC drive liver pathology in NASH (84). Gene manipulated mice for XCR1+ DC (**Table 1**) should be valuable tools for further clarification of *in vivo* roles of cDC1 in various tissues.

## Conclusion

cDC1 were found to be critical in maintaining intestinal immune homeostasis, mainly by supporting intestinal T cell populations. Notably, this homeostatic activity cannot be compensated by the other DC or myeloid lineage cells such as macrophages. At present it remains unclear yet how cDC1 exhibit their functions in the intestine. Commensal bacteria may be involved in the mechanisms, although further studies are still required.

XCR1 is not only a specific marker to cDC1, but also involved in the specific roles of cDC1. Selective expression of XCR1 is observed in various species including mice, rats, sheep, and humans (38, 85, 86). XCL1 is abundantly expressed in NK and activated CD8+ T cells in both humans and mice. The XCR1-XCL1 axis, therefore, seems to be well conserved among species. It would be interesting to clarify whether or how XCR1 or XCR1+ DC function in human intestinal immune homeostasis.

## Author Contributions

All authors were involved in the writing of the manuscript, and read and approved the final version.

## Funding

This work was supported by the Practical Research Project for Rare/Intractable Diseases under Grant Numbers JP19ek0109199 (to HH and TsK), and Grant-in-Aids for Scientific Research (B) (JP26293106, JP17H04088 and JP20H03505 to TsK), for Scientific Research (C) (JP19K07628 to IS, JP18K07071 and JP21K06956 to HH), for Scientific Research on Innovative Areas (JP17H05799 and JP19H04813 to TsK), for Exploratory Research (JP17K19568, JP21K19384 to TsK), for Young Scientists (B) (JP16K19585 and JP18K16096 to YF-O), and for JSPS Research Fellow (JP21J22615 to TaK). This work was also supported by the Uehara Memorial Foundation (to IS and TsK); by Takeda Science Foundation (to IS, HH, and TsK); by the Ichiro Kanehara Foundation for the promotion of Medical Sciences and Medical care (to HH); by the Inamori Foundation (to IS); by GSK Japan Research Grant 2021 (to IS); by Kowa Life Science Foundation (to IS); by the Extramural Collaborative Research Grant of Cancer Research Institute, Kanazawa University; by a Cooperative Research Grant from the Institute for Enzyme Research, Joint Usage/Research Center, Tokushima University; by the Grant for Joint Research Program of the Institute for Genetic Medicine Hokkaido University; by the Grant for Joint Research Project of the Institute of Medical Science, the University of Tokyo; by Wakayama Medical University Special Grant-in-Aid for Research Projects.

## Conflict of Interest

The authors declare that the research was conducted in the absence of any commercial or financial relationships that could be construed as a potential conflict of interest.

## Publisher’s Note

All claims expressed in this article are solely those of the authors and do not necessarily represent those of their affiliated organizations, or those of the publisher, the editors and the reviewers. Any product that may be evaluated in this article, or claim that may be made by its manufacturer, is not guaranteed or endorsed by the publisher.
